# Cooperative Feedback Bits Allocation and Transmit Power Control in Underlay Cognitive Radio Networks

**DOI:** 10.3390/s18061809

**Published:** 2018-06-04

**Authors:** Deokhui Lee, Jaewoo So

**Affiliations:** Department of Electronic Engineering, Sogang University, Seoul 04107, Korea; akirain@sogang.ac.kr

**Keywords:** cognitive radio, limited feedback systems, adaptive feedback bits allocation, transmit power control

## Abstract

In this paper, we consider an underlay cognitive radio network where the spectrum is shared with the primary network. Due to the coexistence of primary and secondary networks, primary users (PUs) are interfered with by the inter-network interference, at the same time secondary users (SUs) counteract the intra-network (inter-user) interference. Based on the cooperative feedback between the primary network and the secondary network, the secondary transmitter (ST) applies the cognitive beamforming to suppress the interference to PUs while improving the sum rate of SUs. We herein propose an adaptive feedback bits allocation among multiple PUs and SUs where the quantized channel direction information (CDI) for the interference channel is forwarded to the ST in order to utilize the beamforming. Moreover, based on the cognitive beamforming, we adjust the transmit power of the ST under the constraint of the average interference at PUs. To jointly solve the feedback bits allocation and the transmit power control problems, we formulate an optimization problem which requires a little iterations compared with the separated feedback bits allocation and the transmit power control problems. Numerical results show that the proposed scheme significantly improves the sum rate of SUs while satisfying the average interference constraint at PUs.

## 1. Introduction

Cognitive radio (CR) has been extensively studied to overcome the spectrum scarcity problem by allowing the secondary users (SUs) to access the spectrum assigned for a primary network [1]. One of the CR strategy, an underlay CR, allows that the primary network and secondary network can transmit signal simultaneously [2,3].

In the underlay CR network, a fundamental challenge is to satisfy the *inter-network interference* constraint from the secondary transmitter (ST) to the primary users (PUs) while improving the performance of the secondary network by minimizing the *intra-network interference* [4,5,6]. Cognitive beamforming and transmit power control are promising techniques that enable the ST to suppress the inter-network interference to PUs [7]. In [8,9], a joint beamforming and power allocation for the CR network is considered to maximize the sum rate of SUs while adjusting the interference to PUs to below a tolerable level. However, the authors of [8,9] assumed the perfect channel direction information (CDI) of the inter-network and intra-network interference channels at the ST. In practical systems, the cognitive beamforming requires the CDI feedback from the primary network to the secondary network, which is called the *cooperative feedback*. Therefore, we focus on communication scenarios where the ST gets quantized CDI via the dedicated feedback channel from PUs and SUs [10].

Many researchers have investigated the effect of the limited feedback on the cognitive beamforming in underlay CR networks. In [11], the authors endeavored to minimize the maximum inter-network interference by adaptively allocating the feedback bits for PUs when the amount of feedback bits for PUs is limited. Nevertheless, PUs may suffer severe interference from the ST because the inter-network interference constraint is not taken into consideration. On the other hands, some researchers have studied how to separate the feedback bits for reporting the CDI and the power control information when the amount of feedback bits per PU is fixed [7,12]. The authors of [7] attempted to minimize the outage probability of serving SUs while satisfying the rate requirement of serving PUs. The authors of [12] aimed to maximize the SU’s link gain under the inter-network interference constraint at the PU. The existing work of [7,11,12] has considered the only cooperative feedback from PUs to the ST in order to mitigate the inter-network interference, whereas the feedback from SUs to the ST is additionally applied to the underlay CR network with the limited feedback so as to enhance the performance of the secondary network by mitigating the intra-network interference [13,14,15]. In [13], authors have calculated the achievable rate of SUs while keeping the interference power at the PU below the predetermined threshold. In [14,15], while the amount of feedback bits is equally allocated to all the PUs, the ST adjusts the transmit power so that the inter-interference becomes less than the predetermined threshold. In [14], the feedback bits allocation scheme was proposed to maximize the sum rate of SUs under the constraint of the amount of feedback bits for SUs. To achieve a balance between the performance of the secondary network and the feedback cost, the authors of [15] proposed the feedback utility function, which is defined as the difference between the average sum rate of SUs and the feedback cost while satisfying the interference constraint at PUs.

Most previous studies equally allocated the feedback bits to PUs (or SUs) or they considered the problem of allocating the total feedback bits among only PUs (or SUs) [7,11,12,13,14,15]. That is, the previous studies dealt with the feedback bits allocation for PUs and SUs separately. Considering that total feedback resource is limited in CR networks; however, it is desired to adaptively adjust the amount of the feedback bits for PUs and SUs. Meanwhile, when the cognitive beamforming is adopted, the transmit power of the ST should be adjusted according to both the interference constraint and the allocated feedback bits for PUs. In previous studies of [7,12,15], the relationship between the transmit power and feedback bits has been investigated under the predetermined interference threshold, but a joint feedback bits allocation and transmit power control scheme has not been proposed.

The contributions of this paper are listed as follows. First, based on the problem of allocating total feedback bits among PUs and SUs, we formulate an optimization problem to maximize the sum rate of SUs while satisfying the average inter-network interference constraint at PUs. We then derive the upper bound of the transmit power of the ST when the amount of feedback bits for PUs is given. Second, to jointly solve the feedback bits allocation and transmit power control problems, we propose an integrated optimization problem in terms of the amount of feedback bits for SUs. In addition, if the proposed iterative algorithm is adopted, it is shown that the number of iterations to find the optimal feedback bits and transmit power of the ST is significantly reduced when compared with separated feedback bits allocation and transmit power control schemes.

The rest of the paper is organized as follows. Section 2 describes the system model and the cooperative feedback procedure. Section 3 presents the joint optimization problem of allocating feedback bits and adjusting the transmit power of the ST, simultaneously. We present the numerical results under various system parameters in Section 4. Finally, Section 5 concludes the paper.

*Notation:* We use bold upper and lower case letters to denote matrices and column vectors, respectively. ${\left(\cdot \right)}^{H}$, ${\left(\cdot \right)}^{T}$, E[·], |·|, and ||·|| respectively denote the conjugate transpose, the transpose, the expectation, the absolute value, and the norm of a vector. The acronym i.i.d. means “independent and identically distributed”.

## 2. System Model

In this section, we describe the system model of the underlay CR network with the cooperative limited feedback.

### 2.1. System Description

**Figure 1:**
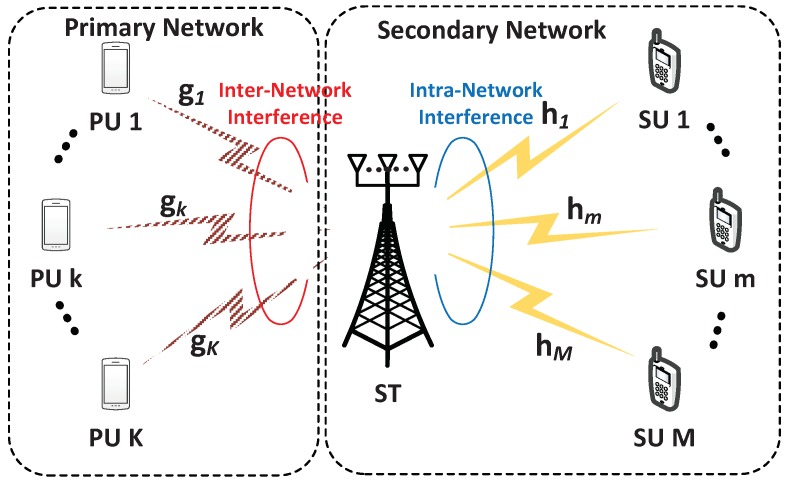
The system model.

We consider an underlay CR network composed of the primary network and the secondary network, as shown in Figure 1. It is assumed that the secondary network shares the same spectrum with the primary network to transmit the data in the downlink. The secondary network includes a ST with $N_{t}$ antennas which serves *M* SUs while satisfying the interference constraint to *K* PUs in the primary network. PUs and SUs are equipped with a single antenna. The interference constraint is the precondition that the primary network allows the secondary network to access the licensed spectrum [15]. Meanwhile, the primary transmitter will also interfere with the SUs. In this paper, we assume that the interference from the primary transmitter to the SU is integrated into the noise at SU. Therefore, we exclude the primary transmitter in the system model.

Let *P* be the transmit power for each SU. It is assumed that the total transmit power at ST is distributed to *M* SUs equally. Then, the received interference signal of PU *k* from ST is represented as

(1) $$y_{pu,k}=\sqrt{PL_{pu,k}}\sum_{m=1}^{M}g_{k}^{H}w_{m}s_{m},$$

where $L_{pu,k}$ is the path loss factor from the ST to the PU *k*; $g_{k}\in C^{N_{t}\times 1}$ is the channel vector from the ST to the PU *k*; $w_{m}\in C^{N_{t}\times 1}$ is the transmit beamforming vector of the ST for SU *m*; and $s_{m}$ denotes the transmitted symbol to SU *m*.

On the other hand, the received signal of SU *m* is expressed as

(2) $$y_{su,m}=\sqrt{PL_{su,m}}h_{m}^{H}w_{m}s_{m}+\sqrt{PL_{su,m}}\sum_{n=1,n\neq m}^{M}h_{m}^{H}w_{n}s_{n}+n_{m},$$

where $L_{su,,m}$ is the path loss factor from the ST to the SU *m*; $h_{m}\in C^{N_{t}\times 1}$ is the channel vector from the BS to CUE *k*; and $n_{m}$ is an additive white Gaussian noise (AWGN) for SU *m* with zero mean and unit variance. It is assumed that all the channel elements are drawn from i.i.d. complex Gaussian random variables with zero mean and unit variance. Please note that the first term in Equation (2) accounts for the desired signal, while the second term denotes the intra-network interference signal.

Then, the instantaneous interference from the ST to PU *k* is given by

(3) $$I_{pu,k}=PL_{pu,k}\sum_{m=1}^{M}{\left|g_{k}^{H}w_{m}\right|}^{2}.$$

As the interference constraint, the average interference constraint and the peak interference constraint are commonly adopted for the constraint condition in the CR system. As shown in the previous works of [16,17], The average interference constraint is more favorable than the peak interference constraint in aspects of the throughput maximization. Accordingly, we assume the average interference constraint for the underlay CR network. Let $I_{th}$ be the allowable maximum average interference from the ST to PUs. Then, the average inter-network interference constraint is defined by

(4) $$E\left[I_{pu,k}\right]\leq I_{th}.$$

Meanwhile, the instantaneous SNR of the SU *m* is represented by

(5) $$\gamma_{m}=\frac{PL_{su,m}{\left|h_{m}^{H}w_{m}\right|}^{2}}{1+PL_{su,m}\sum_{n=1,n\neq m}^{M}{\left|h_{m}^{H}w_{n}\right|}^{2}},$$

where the intra-network interference at SU *m* is defined by $I_{su,m}=PL_{su,m}\sum_{n=1,n\neq m}^{M}{\left|h_{m}^{H}w_{n}\right|}^{2}$.

### 2.2. Cooperative Limited Feedback

We consider the cooperative limited feedback between the primary network and the secondary network. The secondary network purchases the *partial* feedback resource to obtain the CDI of the interference channel from the ST to PUs. We herein focus on the quantization procedure for the CDI of the inter-network and intra-network interference channels, which are defined as ${\tilde{g}}_{k}=g_{k}/\left|\right|g_{k}\left|\right|$ and ${\tilde{h}}_{m}=h_{m}/\left|\right|h_{m}\left|\right|$, respectively.

In the cooperative limited feedback system, each user firstly quantizes the CDI of the received interference channel through the given codebooks designed by the random vector quantization (RVQ) method [18,19]. Let us define the codebook for the ST, the PU *k*, and the SU *m* as

(6) $$\begin{array}{ccc} C_{pu,k} & = & \{c_{pu,k}^{\ell }|\ell =1,\cdots ,2^{\beta_{pu,k}}\}, \end{array}$$

(7) $$\begin{array}{ccc} C_{su,m} & = & \{c_{su,m}^{\ell }|\ell =1,\cdots ,2^{\beta_{su,m}}\}, \end{array}$$

where $c_{pu,k}^{\ell }\in C^{N_{t}\times 1}$ denotes the *ℓ*th codeword for the ST and the PU *k*; $\beta_{pu,k}$ indicates the number of allocated feedback bits between the ST and the PU *k*; $c_{su,m}^{\ell }\in C^{N_{t}\times 1}$ denotes the *ℓ*th codeword for the ST and the SU *m*; and $\beta_{su,m}$ indicates the number of allocated feedback bits between the ST and the SU *m*; Then, the quantized CDI is obtained as

(8) $$\begin{array}{ccc} {\hat{g}}_{k} & = & \arg\max_{c_{pu,k}^{\ell }\in C_{pu,k}}\left|{\tilde{g}}_{k}c_{pu,k}^{\ell }\right|, \end{array}$$

(9) $$\begin{array}{ccc} {\hat{h}}_{m} & = & \arg\max_{c_{su,m}^{\ell }\in C_{su,m}}\left|{\tilde{h}}_{m}c_{su,m}^{\ell }\right|. \end{array}$$

Then, each PU and SU feeds back the codeword indices to the ST without loss and delay. We herein consider that the total number of feedback bits for the PUs and SUs is limited to

(10) $$\begin{array}{ccc} \beta^{T} & = & \sum_{k=1}^{K}\beta_{pu,k}+\sum_{m=1}^{M}\beta_{su,m}\\ & = & \beta_{pu}^{T}+\beta_{su}^{T}, \end{array}$$

where $\beta_{pu}^{T}$ and $\beta_{su}^{T}$ are the sum of the allocated feedback bits for PUs and SUs, respectively. It is assumed that the feedback bits for the channel quality information (CQI) is not included in $\beta^{T}$ bits. In other words, to focus on the effect of the quantized CDI with the cognitive beamforming, we assumed that the *k*th PU and the *m*th SU can perfectly report its CQI to the ST, i.e., $\left|\right|g_{k}{\left|\right|}^{2}$ and $\left|\right|h_{m}{\left|\right|}^{2}$.

Based on the quantized CDI feedback from PUs and SUs to the ST, we utilize the transmit zero-forcing beamforming (ZFBF) to mitigate the inter-network and intra-network interference, simultaneously. The transmit beamforming vector for the SU *m* is determined by

(11) $${\left[\hat{G},{\hat{H}}_{m}\right]}^{H}w_{m}=0,$$

where the set of the inter-network interference channels is denoted by $\hat{G}$ which is defined as $\hat{G}=\left[\cdots ,{\hat{g}}_{k},\cdots \right]$ for k=1,⋯,K; and the set of the intra-network interference channels for SU *m* is ${\hat{H}}_{m}$ which is defined as ${\hat{H}}_{m}=\left[\cdots ,{\hat{h}}_{n},\cdots \right]$ for n=1,⋯,M and n≠m.

## 3. Proposed Joint Feedback Bits Allocation and Transmit Power Control

In this section, we propose a joint feedback bits allocation and the transmit power control to maximize the sum rate of the secondary network while satisfying the average interference constraint from the ST to PUs. Intuitively, when $I_{th}$ is given, allocating more feedback bits for PUs can inform more accurate quantized CDI to the ST, which results in the reduction of the inter-network interference. At the same time, the available transmit power of the ST increases until satisfying the average interference constraint; however, the intra-network interference also increases because the number of available feedback bits for SUs is reduced due to the limitation of the total feedback bits for the CR network. Accordingly, to maximize the sum rate of SUs, we should achieve a balance between the transmit power and the allocation of the feedback bits between PUs and SUs.

### 3.1. Transmit Power Control and Feedback Bits Allocation for PUs

When the number of feedback bits for PU is given, we determine the transmit power so that the inter-network interference to the PU meets the allowable average interference constraint. The average interference from the ST to the PU *k* can be rewritten as follows:

(12) $$E\left[I_{pu,k}\right]=PL_{pu,k}E[||g_{k}{\left|\right|}^{2}]\sum_{m=1}^{M}E[|{\tilde{g}}_{k}^{H}w_{m}{|}^{2}].$$

If the obtained CDI at the ST is perfect, $E[{\tilde{g}}_{k}^{H}w_{m}]$ of (12) becomes zero by the beamforming. However, the obtained CDI at the ST is imperfect due to the quantization, and thus the interference is residual to the PU. Therefore, we investigated the relationship between the average interference and the number of assigned feedback bits.

**Theorem** **1.** *Given $\beta_{pu,k}$, the average interference from the ST to the PU k is tightly upper bounded by*

(13) $$E\left[I_{pu,k}\right]<PL_{pu,k}MN2^{\frac{-\beta_{pu,k}}{N_{t}-1}},$$

*where we connote $N=N_{t}/\left(N_{t}-1\right)$ for the notational simplicity.*

**Proof** **of** **Theorem** **1.** According to the theorem of RVQ, we decompose the channel vector as

(14) $${\tilde{g}}_{k}=\sqrt{1-\kappa }{\hat{g}}_{k}+\sqrt{\kappa }e_{k},$$

where κ = $\sin^{2}\left(∠\left({\tilde{g}}_{k},{\hat{g}}_{k}\right)\right)$ is the amplitude of the quantization error; and $e_{k}$ is an i.i.d unit norm vector. Since the transmit beamforming vector based on the quantized CDI is designed to null out the interference, we can obtain

(15) $$|{\tilde{g}}_{k}^{H}w_{m}{|}^{2}=\kappa {\left|e_{k}^{H}w_{m}\right|}^{2}.$$

Then, by using the fact that $E\left[\kappa \right]<2^{-\beta_{pu,k}/\left(N_{t}-1\right)}$ and $E[|e_{k}^{H}w_{m}{|}^{2}]=1/\left(N_{t}-1\right)$ [19], we can easily represent the upper bound of the average interference from the ST to the PU *k* as Equation (13). ☐

Let $P_{k}$ be the transmit power for each SU to satisfy the interference constraint, $I_{th}$ for PU *k* when $\beta_{pu,k}$ is given. Then, substituting Equation (13) with Equation (4), the upper bound of *P* can be represented by

(16) $$P_{k}≜P=\frac{I_{th}}{L_{pu,k}MN}2^{\frac{\beta_{pu,k}}{N_{t}-1}}.$$

Please note that the obtained $P_{k}$ is a valid transmit power for only PU *k*.

Next, suppose that the transmit power of the ST for each SU is *P*. Then, the feedback bits allocation for PU *k* can be easily determined. We rearrange Equation (16) in terms of $\beta_{pu,k}$. As a result, we obtain the optimal number of feedback bits for each PU *k*, $\beta_{pu,k}^{*}$, which is represented by

(17) $$\beta_{pu,k}^{*}={\left\lfloor \left(N_{t}-1\right)\log_{2}\left(\frac{PL_{pu,k}MN}{I_{th}}\right)\right\rfloor }^{+},$$

where ${\left\lfloor x\right\rfloor }^{+}=\max\left\{0,\left\lfloor x\right\rceil \right\}$ and ⌊x⌉ indicates the round operation.

Now, when *P* is given, we present the optimal feedback bits for each PU as shown in Equation (17). Then, the optimal amount of feedback bits for PUs is expressed as

(18) $$\begin{array}{ccc} \beta_{pu}^{T} & = & \sum_{k=1}^{K}{\left\lfloor \left(N_{t}-1\right)\log_{2}\left(\frac{PL_{pu,k}MN}{I_{th}}\right)\right\rfloor }^{+} \end{array}$$

(19) $$\begin{array}{cc} \;\;\;\;\;\;= & \left(N_{t}-1\right)\left[\log_{2}{\left(\frac{PMN}{I_{th}}\right)}^{K}+\log_{2}\left(\prod_{k=1}^{K}L_{pu,k}\right)\right], \end{array}$$

where we imply that the round operation in Equation (18) can be removed. We rearrange Equation (19) in terms of *P*, and we obtain the optimal transmit power of the ST, $P^{*}$, as follows:

(20) $$\begin{array}{ccc} P^{*} & = & \frac{I_{th}}{MN}{\left(\prod_{k=1}^{K}L_{pu,k}\right)}^{-1/K}2^{\frac{\beta_{pu}^{T}}{K(N_{t}-1)}}\\ & = & \frac{I_{th}}{MN}{\left(\prod_{k=1}^{K}L_{pu,k}\right)}^{-1/K}2^{\frac{\beta^{T}-\beta_{su}^{T}}{K(N_{t}-1)}}. \end{array}$$

Interestingly, it is confirmed that the optimal transmit power is adjusted according to the amount of feedback bits for PUs or SUs, i.e., $\beta_{pu}^{T}$ or $\beta_{su}^{T}$.

### 3.2. Feedback Bits Allocation for SUs

In this subsection, when the amount of feedback bits for SUs is given, we propose the feedback bits allocation scheme to maximize the sum rate of SUs. Before allocating the feedback bits for SUs, we investigate the average rate loss between the perfect CDI and the quantized CDI feedback at the secondary network. It is well-known that the problem minimizing the average rate loss is the alternative optimization problem maximizing the data rate when the transmit power is given in the limited feedback system [20,21].

Let $▵r_{m}$ be the average rate loss of the SU *m*, and we have

(21) $$\begin{array}{ccc} ▵r_{m} & ≜ & E\left[\log_{2}\left(1+PL_{su,m}\left|\right|h_{m}{\left|\right|}^{2}\right)\right]-E\left[\log_{2}\left(1+\frac{PL_{su,m}{\left|h_{m}^{H}w_{m}\right|}^{2}}{1+I_{su,m}}\right)\right]\\ & = & \underset{{▵}_{m}}{\underset{︸}{E\left[\log_{2}\left(\frac{1+PL_{su,m}\left|\right|h_{m}{\left|\right|}^{2}}{1+PL_{su,m}{\left|h_{m}^{H}w_{m}\right|}^{2}+I_{su,m}}\right)\right]}}+E\left[\log_{2}\left(1+I_{su,m}\right)\right]\\ & \leq & \log_{2}\left(1+E\left[I_{su,m}\right]\right), \end{array}$$

where $\log_{2}(1+PL_{su,m}\left|\right|h_{m}{\left|\right|}^{2})$ term is the data rate with the perfect CDI which perfectly nulls out the intra-network interference. The inequality is obtained by setting ${▵}_{m}\leq 0$ and applying Jensen’s inequality [22]. According to the *Theorem 1*, the average intra-network interference among the SUs is tightly upper bounded by

(22) $$\begin{array}{ccc} E[I_{su,m}] & = & PL_{su,m}E[||h_{m}{\left|\right|}^{2}]\sum_{n=1,n\neq m}^{M}E[|{\tilde{h}}_{m}^{H}w_{n}{|}^{2}]\\ & < & PL_{su,m}\left(M-1\right)N2^{\frac{-\beta_{su,m}}{N_{t}-1}}. \end{array}$$

Then, the average sum rate loss of the SUs is bounded as

(23) $$\begin{array}{ccc} \sum_{m=1}^{M}\log_{2}\left(1+E\left[I_{su,m}\right]\right) & \leq & \sum_{m=1}^{M}\log_{2}\left(1+PL_{su,m}\left(M-1\right)N2^{\frac{-\beta_{su,m}}{N_{t}-1}}\right)\\ & = & \log_{2}\left\{\prod_{m=1}^{M}\left(1+PL_{su,m}\left(M-1\right)N2^{\frac{-\beta_{su,m}}{N_{t}-1}}\right)\right\}. \end{array}$$

Based on the upper bound of the average sum rate of SUs, we can formulate the feedback bits allocation problem for SUs as follows.

(24) $$\begin{array}{cc} \min & \prod_{m=1}^{M}\left(1+PL_{su,m}\left(M-1\right)N2^{\frac{-\beta_{su,m}}{N_{t}-1}}\right)\\ s.t. & \sum_{m=1}^{M}\beta_{su,m}\leq \beta_{su}^{T}. \end{array}$$

The optimization problem of Equation (24) is a logarithmically convex function and therefore we can find the optimal solution. The Lagrangian function of Equation (24) is given by

(25) $$L\left(\beta_{su,m},\lambda \right)=\prod_{m=1}^{M}\left(1+PL_{su,m}\left(M-1\right)N2^{\frac{-\beta_{su,m}}{N_{t}-1}}\right)+\lambda \left(\sum_{m=1}^{M}\beta_{su,m}-\beta_{su}^{T}\right),\;\forall m,$$

where λ is the Lagrangian multiplier for the constraint. Applying the Karush-Kuhn-Tucker (KKT) condition, we can obtain the following necessary and sufficient conditions,

(26) $$\frac{L\left(\beta_{su,m},\lambda \right)}{\partial \beta_{su,m}}=\frac{-\ln\left(2\right)PL_{su,m}\left(M-1\right)N}{\left(N_{t}-1\right)}2^{\frac{-\beta_{su,m}}{N_{t}-1}}\prod_{n=1,n\neq m}^{M}\left(1+PL_{su,n}\left(M-1\right)N2^{\frac{-\beta_{su,n}}{N_{t}-1}}\right)+\lambda =0$$

for m=1,⋯,M. Additionally, we have

(27) $$\frac{L\left(\beta_{su,m},\lambda \right)}{\partial \lambda }=\sum_{m=1}^{M}\beta_{su,m}-\beta_{su}^{T}=0.$$

By substituting Equation (26) into Equation (27), we obtain the optimal number of allocated feedback bits for SU *m* as follows:

(28) $$\beta_{su,m}^{*}={\left\lfloor \beta_{su}^{T},\;\frac{\beta_{su}^{T}}{M}+\left(N_{t}-1\right)\log_{2}\left(\frac{PL_{su,m}N}{\prod_{n=1}^{M}{\left(PL_{su,n}N\right)}^{1/M}}\right)\right\rfloor }^{+},\;\forall \;m.$$

Even though the feedback bits allocation strategy is based on the upper bound of the average rate loss, we notice the following information. The allocated feedback bits for each channel link depend on the number of transmit antennas, $N_{t}$, the number of serving SUs, *M*, and the geometric mean of the total received power by the intra-network interference. Hence, more feedback bits are allocated to the stronger channel link to minimize the average rate loss.

### 3.3. Iterative Feedback Bits Allocation and Transmit Power Control

In Section 3.1, the transmit power control and feedback bits allocation for PUs are investigated, whereas in Section 3.2, the feedback bits allocation for SUs is proposed when the transmit power of the ST is given. However, the feedback bits allocation and the transmit power control problems should be jointly solved to maximize the sum rate of SUs while satisfying the average interference constraint.

We first derive the lower bound of the average data rate of SU *m*. From the result (22), we have

(29) $$\begin{array}{ccc} E\left[\log_{2}\left(1+\frac{PL_{su,m}{\left|h_{m}^{H}w_{m}\right|}^{2}}{1+I_{su,m}}\right)\right] & \geq & \log_{2}\left(\frac{1+PL_{su,m}E[||h_{m}{\left|\right|}^{2}]}{1+E[I_{su,m}]}\right)\\ & \geq & \log_{2}\left(\frac{1+PL_{su,m}N_{t}}{1+PL_{su,m}\left(M-1\right)N2^{\frac{-\beta_{su,m}}{N_{t}-1}}}\right),\;\forall m. \end{array}$$

Then, the joint optimization problem of the feedback bits allocation and transmit power control can be written as

(30) $$\begin{array}{cc} \max & \sum_{m=1}^{M}\log_{2}\left(\frac{1+PL_{su,m}N_{t}}{1+PL_{su,m}\left(M-1\right)N2^{\frac{-\beta_{su,m}}{N_{t}-1}}}\right)\\ s.t. & \left(C1\right)\;\;PL_{pu,k}MN2^{\frac{-\beta_{pu,k}}{N_{t}-1}}\leq I_{th},\;\forall k,\\ & \left(C2\right)\;\;\sum_{k=1}^{K}\beta_{pu,k}+\sum_{m=1}^{M}\beta_{su,m}\leq \beta^{T},\\ & \left(C3\right)\;\;P\leq P_{\max}, \end{array}$$

where (C1) is the average interference constraint at PUs; (C2) is the total number of feedback bits constraint; and (C3) is the maximum allowed transmit power constraint for each SU.

To solve the joint optimization problem, we integrate the object function (30) with the result of the optimal transmit power of the ST in (20) and the feedback bits allocation for SUs in (28). We first substitute (28) into the object function which stands for maximizing the sum rate of SUs when *P* and $\beta_{su}^{T}$ are given. Then, *P* of the object function is replaced with (20), where the substituted *P* satisfies the average interference constraint at PUs when $\beta_{su}^{T}$ is given. After the above mathematical calculations, the object function is expressed as the function of $\beta_{su}^{T}$, which is represented by

(31) $$\sum_{m=1}^{M}F_{m}\left(\beta_{su}^{T}\right)=\sum_{m=1}^{M}\log_{2}\left(1+\mu L_{su,m}2^{\frac{-\beta_{su}^{T}}{K(N_{t}-1)}}\right)-\log_{2}\left(1+\nu 2^{\frac{-\left(K+M\right)\beta_{su}^{T}}{KM(N_{t}-1)}}\right),$$

$$\begin{array}{ccc} \mu & = & \frac{I_{th}\left(N_{t}-1\right)}{M}{\left(\prod_{k=1}^{K}L_{pu,k}\right)}^{-1/K}2^{\frac{\beta^{T}}{K(N_{t}-1)}},\\ \nu & = & \frac{I_{th}\left(M-1\right)}{M}{\left(\prod_{k=1}^{K}L_{pu,k}\right)}^{-1/K}{\left(\prod_{m=1}^{M}L_{su,m}\right)}^{1/M}2^{\frac{\beta^{T}}{K(N_{t}-1)}}, \end{array}$$

where we denote some positive constants by using μ and ν for the sake of clarity. As shown in Equation (31), the data rate of SU *m* is determined by $\beta_{su}^{T}$ when the proposed feedback bits allocation and transmit power control are adopted. Accordingly, finding the optimal $\beta_{su}^{T}$ plays a key role in maximizing the sum rate of SUs while satisfying the average interference constraint at PUs.

We now investigate the iterative algorithm to find the optimal $\beta_{su}^{T}$. The iterative algorithm is based on the bisection method. Firstly, taking the derivative of $F_{m}\left(\beta_{su}^{T}\right)$ with respect to $\beta_{su}^{T}$, we have

(32) $$\frac{\partial F_{m}\left(\beta_{su}^{T}\right)}{\partial \beta_{su}^{T}}=\frac{-\mu L_{su,m}}{K\left(N_{t}-1\right)\left(1+\mu L_{su,m}2^{\frac{-\beta_{su}^{T}}{K(N_{t}-1)}}\right)}2^{\frac{-\beta_{su}^{T}}{K(N_{t}-1)}}+\frac{(K+M)\nu }{KM\left(N_{t}-1\right)\left(1+\nu 2^{\frac{-\left(K+M\right)\beta_{su}^{T}}{KM(N_{t}-1)}}\right)}2^{\frac{-\left(K+M\right)\beta_{su}^{T}}{KM(N_{t}-1)}},$$

for m=1,⋯,M. Then, according to the value of $\sum_{m=1}^{M}\partial F_{m}\left(\beta_{su}^{T}\right)/\partial \beta_{su}^{T}$, we varies $\beta_{su}^{T}$ until the value of $\sum_{m=1}^{M}\partial F_{m}\left(\beta_{su}^{T}\right)/\partial \beta_{su}^{T}$ is converged. The iterative algorithm is outlined as follows:

**Algorithm 1** Iterative algorithm to find the optimal $\beta_{su}^{T}$
1:Initialize $\beta_{su}^{UB}=\beta^{T}$ and $\beta_{su}^{LB}=\beta^{T}-\beta_{pu}^{T}$, where $\beta_{pu}^{T}$ is determined by Equation (19) when $P=P_{\max}$2:Set $\beta_{su}^{T}\leftarrow {\left\lfloor \left(\beta_{su}^{UB}+\beta_{su}^{LB}\right)/2\right\rfloor }^{+}$3:**while**$\left(\beta_{su}^{UB}\neq \beta_{su}^{T}\right)$ and $\left(\beta_{su}^{LB}\neq \beta_{su}^{T}\right)$ **do**4:  **if** $\sum_{m=1}^{M}\partial F_{m}\left(\beta_{su}^{T}\right)/\partial \beta_{su}^{T}>0$ **then**5:    Update $\beta_{su}^{LB}\leftarrow \beta_{su}^{T}$6:  **else if** $\sum_{m=1}^{M}\partial F_{m}\left(\beta_{su}^{T}\right)/\partial \beta_{su}^{T}<0$ **then**7:    Update $\beta_{su}^{UB}\leftarrow \beta_{su}^{T}$8:  **end if**9:  Update $\beta_{su}^{T}\leftarrow {\left\lfloor \left(\beta_{su}^{UB}+\beta_{su}^{LB}\right)/2\right\rfloor }^{+}$10:
**end while**
11:
**return** $\beta_{su}^{T}$



Let the lower bound and upper bound for the sum of feedback bits for SUs be respectively denoted by $\beta_{su}^{LB}$ and $\beta_{su}^{UB}$. We first initialize $\beta_{su}^{LB}$, $\beta_{su}^{UB}$ and $\beta_{su}^{T}$. We then find optimal $\beta_{su}^{T}$ by using the bisection method until any of $\beta_{su}^{LB}$ and $\beta_{su}^{UB}$ equals to $\beta_{su}^{T}$. Since the amount of feedback bits has an integer value, the bisection method requires $\left\lceil \log_{2}\left(\beta_{su}^{UB}-\beta_{su}^{LB}\right)\right\rceil$ iterations to converge $\beta_{su}^{UB}-\beta_{su}^{LB}\leq 1$ [23,24].

Meanwhile, we investigate the number of required iterations when the feedback bits allocation and transmit power control problems are separately solved. Let the lower bound and the upper bound for the transmit power of the ST be the $P^{LB}$ and $P^{UB}$, respectively. Then, the bisection method requires $\left\lceil \log_{2}\left(P^{UB}-P^{LB}\right)/\epsilon \right\rceil$ iterations to converge $P^{UB}-P^{LB}<\epsilon$, where ϵ is the accuracy. Since ϵ is a small value and $P^{UB}-P^{LB}$ is significantly bigger than $\beta_{su}^{UB}-\beta_{su}^{LB}$, the separated feedback bits allocation and transmit power control problems require more iterations compared with the joint optimization problem.

## 4. Numerical Results

In this section, we present the numerical results to validate the proposed feedback bits allocation and transmit power control scheme. We consider the underlay CR network with K=2, M=2, and $N_{t}=4$. Let $d_{su,m}$ be the distance between the ST and SU *m*; and $d_{pu,k}$ be the distance between the ST and PU *k*. We define the average received signal power from the ST to the SU *m* as

(33) $${\bar{P}}_{su,m}=PL_{su,m},$$

where $L_{su,m}={\left(d_{0}/d_{su,m}\right)}^{\alpha }$. The path loss factor for PU *k* is defined as $L_{pu,k}={\left(d_{0}/d_{pu,k}\right)}^{\alpha }$ in a similar manner. We set the reference distance to be unity, i.e., $d_{0}=1$ m. The path loss coefficient is considered as α=3.8. For all scenarios, $P_{\max}$ is set to be ${\bar{P}}_{su,m}=0$ dB at the cell edge, $d_{su,m}=500$ m. Table 1 summarizes parameters used for numerical results.

For the performance comparison, we consider two conventional schemes that allocate $\beta_{su}^{T}\left(\;=\;\beta^{T}-\beta_{pu}^{T}\right)$ bits for SUs. In the the equal feedback bits allocation (EFA) scheme, the amount of feedback bits is equally allocated to all SUs, i.e., $\beta_{su,m}=\beta_{su}^{T}/M$, ∀m. In the adaptive feedback bits allocation (AFA) scheme based on Equation (28), the amount of feedback bits is adaptively allocated for each SU to maximize the sum rate of SUs. For the cooperative feedback, it is assumed that the amount of feedback bits is equally allocated for all PUs, i.e., $\beta_{pu,k}\;=\;\beta^{T}/\left(K+M\right)$, ∀k. Then, the transmit power of the ST is determined by $\min(P_{k})$, ∀k. The above strategy is similar with the conventional scheme of [15]. In addition, the overall numerical results are obtained using 10,000 independent PU and SU drop events.

**Figure 2:**
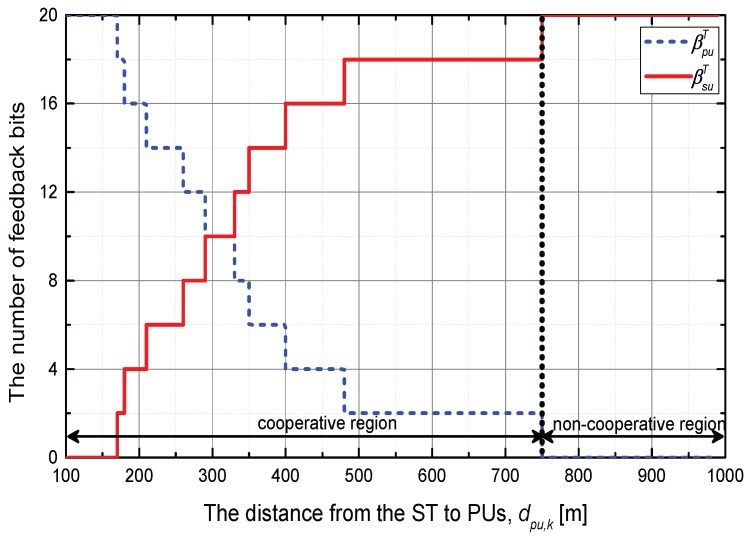
Feedback bits allocation strategy when SUs are located at $d_{su,m}=250$ m.

First of all, we investigate the feedback bits allocation and transmit power control strategy according to the distance between the ST and PUs when $d_{su,m}=250$ m for SUs. Here, it is assumed that $\beta^{T}$ is set as 20 bits; and $I_{th}$ is fixed to 0 dB. The feedback bits allocation strategy is presented in Figure 2. For the *cooperative feedback region*, $d_{pu,k}=\left[0,750\right]$ m, as $\beta_{pu,k}$ decreases, a more accurate CDI for the inter-network interference channel is required to satisfy the average interference constraint at PUs. Meanwhile, in a *non-cooperative feedback region*, $d_{pu,k}=\left[750,1000\right]$ m, the CDI of the inter-network interference channel is not necessary since the strength of the interference signal is sufficiently weak. The obtained region can be exploited to decide whether the cooperative feedback is performed or not.

At the same time, the power allocation strategy is shown in Table 2. We presented ${\bar{P}}_{su,m}$ at the cell edge according to the optimal *P*. As $d_{pu,k}$ increases, ${\bar{P}}_{su,m}$ converges to 0 dB, which means that *P* reaches the $P_{\max}$ since the *P* is scaled as the product of the geometric mean of the inverse of the path loss factor, i.e., ${\left(\prod_{k=1}^{K}L_{pu,k}\right)}^{-1/K}$ by Equation (20).

**Figure 3:**
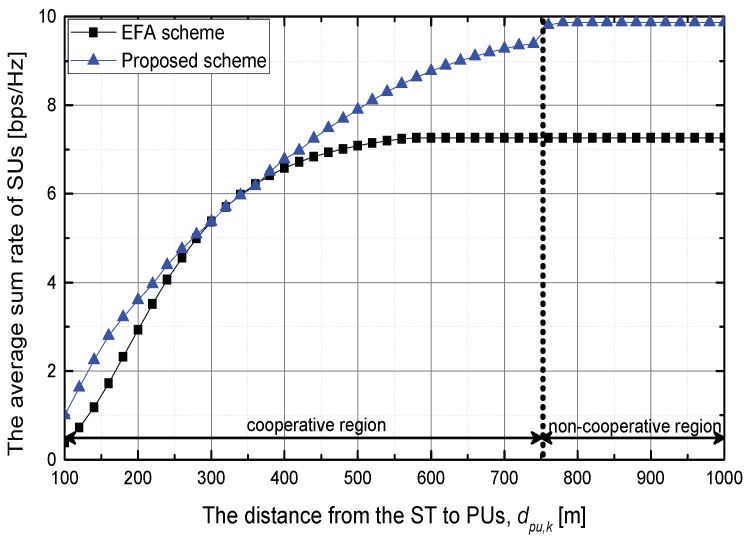
The average sum rate of SUs when SUs are located at $d_{su,m}=250$ m.

Figure 3 shows the average sum rate of SUs in the above scenario. In the cooperative region, the average sum rate logarithmically increases because the intra-network interference signal decreases. At the region $d_{pu,k}=\left[0,300\right]$ m, the proposed scheme slightly increases the average sum rate of SUs compared with the EFA scheme because the strength of the inter-network interference signal is too strong. As $d_{pu,k}$ increases, the proposed scheme allocates more feedback bits to SUs, and increases the performance compared with the EFA scheme. When $d_{pu,k}=740$ m, the proposed scheme increases the sum rate by about 29.1% in comparison with the EFA scheme. In the non-cooperative region, the average sum rate of SUs converges since the total feedback bits is allocated for SUs and the maximum transmit power of the ST is used.

**Figure 4:**
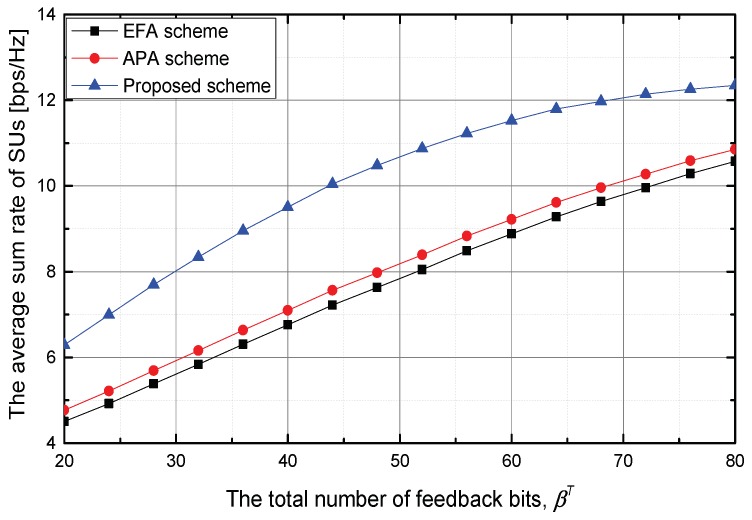
The average sum rate of SUs versus the total number of feedback bits, $\beta^{T}$.

Next, we investigate the performance of the proposed scheme according to the total number of feedback bits when $I_{th}$ is fixed to 0 dB. In this scenario, $d_{pu,k}$ is uniformly distributed between 100 m and 500 m; and $d_{su,k}$ is uniformly distributed between 100 m and 750 m, where this range is the cooperative region as shown in Figure 2. Figure 4 shows the average sum rate of SUs. It is confirmed that the proposed scheme outperforms the EFA and AFA schemes. This is due to the fact that the proposed scheme adaptively allocates the total feedback bits between PUs and SUs. Moreover, in the proposed scheme, the transmit power of the ST is adjusted to maximize the average sum rate of SUs while satisfying the average interference constraint at PUs. When $\beta^{T}=32$ bits, the proposed scheme increases the average sum rate of SUs by about 35.3% and 42.8% in comparison with the EFA and AFA schemes, respectively. Meanwhile, although the AFA scheme adaptively allocates the feedback bits for SUs, it increases slightly the sum rate of SUs compared with the EFA scheme.

**Figure 5:**
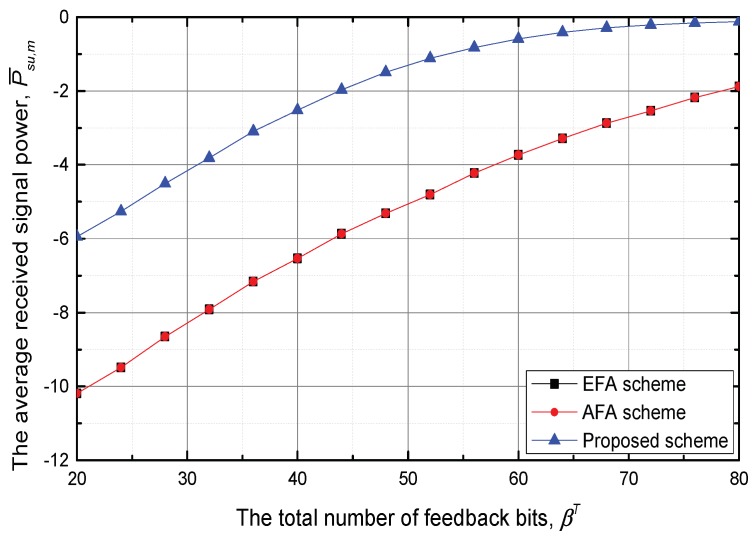
The average received signal power at the cell edge, ${\bar{P}}_{su,m}$ versus the total number of feedback bits, $\beta^{T}$.

Figure 5 shows the average received signal power of the SU at the cell edge. As value of $\beta^{T}$ increases, *P* converges to the $P_{\max}$ since the number of allocated feedback bits for PUs increases. Since the feedback bits are dynamically allocated to PUs, the proposed scheme can mitigate the inter-network interference compared with the EFA and AFA schemes. This fact facilitates that the available *P* of the proposed scheme is greater than those of the EFA and AFA schemes.

**Figure 6:**
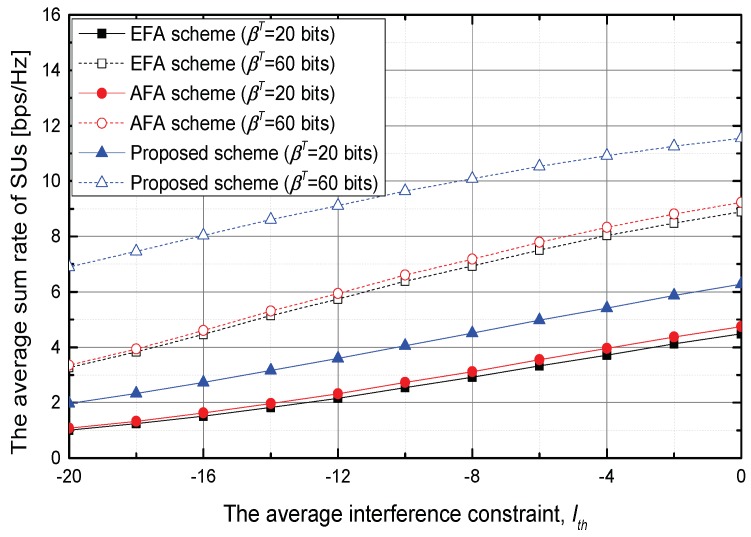
The average sum rate of SUs versus the average interference constraint, $I_{th}$.

Finally, Figure 6 shows the average sum rate of SUs according to the variations of the $I_{th}$. As $I_{th}$ relaxes, the average sum rate of SUs becomes increases. In aspects of the average sum rate of SUs while satisfying the average interference constraint, it is a meaningful result that the proposed scheme outperforms the EFA and AFA schemes for the different values of $I_{th}$ and $\beta^{T}$. When $I_{th}=-20$ dB and $\beta^{T}=20$ bits, the proposed scheme increases the average sum rate of SUs by about 96.5% and 83.8% compared with the EFA and AFA scheme, respectively.

## 5. Conclusions

In this paper, we proposed a joint feedback bits allocation and transmit power control scheme when the total number of feedback bits is limited for CR networks. Specifically, based on the cooperative feedback from PUs to the ST, the cognitive beamforming is adopted to suppress the inter-network and intra-network interference, simultaneously. We formulated the integrated optimization problem, which represents the sum rate of SUs while satisfying the average interference constraint at PUs. Furthermore, the problem of maximizing the sum rate of SUs is easily solved by the iteration algorithm, which requires a little iterations compared with the separated feedback bits allocation and transmit power control problems. Numerical results are presented under the various scenarios of the total number of feedback bits, the average interference constraint, and the various user’s location. Finally, the numerical results show that the proposed scheme significantly improves the sum rate of SUs compared with conventional schemes.

## Figures and Tables

**Table 1:** Parameters for numerical results.

Parameter	Value
The number of PUs, *K*	2
The number of SUs, *M*	2
The number of transmit antennas at ST, $N_{t}$	4
The path loss coefficient, α	3.8
The reference distance, $d_{0}$	1 m
The average received signal power, ${\bar{P}}_{su,m}$ at $d_{su,m}=500$ m	0 dB

**Table 2:** Transmit power control strategy when SUs are located at $d_{su,m}=250$ m.

$d_{pu,k}$	100 m	200 m	300 m	400 m	500 m	600 m	700 m	800 m	900 m
${\bar{P}}_{su,m}$	−23.8 dB	−14.4 dB	−10.7 dB	−7.9 dB	−6.3 dB	−3.3 dB	−0.7 dB	0 dB	0 dB
